# *Ex* *vivo* susceptibility to dihydroartemisinin in *Plasmodium falciparum* patient isolates from Eastern Rwanda, 2025

**DOI:** 10.1186/s12936-026-06107-4

**Published:** 2026-08-19

**Authors:** Rafael Oliveira, Jean Pierre Musabyimana, Emmanuel Kabalisa, Pierre Gashema, Fabien Nshimiyimana, Jean Damascene Buregeya, Immaculee Uwumutima, Eugene Hitimana, Felix Habarugira, Christian Ngarambe, Claude Mambo Muvunyi, Jules Ndoli Minega, Frank P. Mockenhaupt, Welmoed van Loon

**Affiliations:** 1 Institute of International Health, Charité Center for Global Health, Charité - Universitaetsmedizin Berlin, Berlin, Germany; 2 Rwanda Biomedical Center, Kigali, Rwanda; 3 Kabutare District Hospital, Huye, Rwanda; 4 National Reference Laboratory, Rwanda Biomedical Center, Kigali, Rwanda; 5 Clinical Education and Research Division, University Teaching Hospital of Butare, Butare, Rwanda

**Keywords:** Artemisinin resistance, PfK13, *Ex vivo*, RSA, eRRSA, Rwanda

## Abstract

**Background:**

The emergence of artemisinin partial resistance (AR) in *Plasmodium falciparum*, characterized by mutations in the *Plasmodium falciparum Kelch 13* (*PfK13*) gene and delayed parasite clearance, represents a significant threat to malaria control in Africa. While the Ring-Stage Survival Assay (RSA) is the phenotypic gold standard for AR detection, its technical complexity limits scalability. The Extended Recovery Ring-Stage Survival Assay (eRRSA), utilizing quantitative PCR (qPCR), has been proposed as a more scalable alternative for molecular surveillance.

**Methods:**

In early 2025, 19 clinical *P. falciparum* isolates were collected and analyzed from the Kirehe district of Rwanda. AR phenotypes were characterized using both ex vivo RSA (72-h microscopy) and eRRSA (120-h qPCR). Parasite genotypes were determined via Sanger sequencing of the *PfK13* propeller domain. Statistical analyses, including Spearman correlation and Receiver Operating Characteristic (ROC) curves, were applied to compare assay performance and define resistance thresholds.

**Results:**

Non-synonymous *PfK13* mutations were identified in 52.6% (10/19) of isolates, predominantly the validated R561H marker (31.6%). The N490T mutation (2/19) was detected for the first time in Rwanda. RSA-confirmed AR (survival rate >1%) was present in 38.9% of valid assays. The R561H mutation was significantly associated with elevated RSA survival (*P*=0.02), and the N490T isolates had high survival rates. Two further rare variants, P667S (2/18) and F699C (1/18), did not show elevated survival rates. eRRSA recovery rates correlated with RSA survival (*P*=0.003, ρ=−0.7); however, eRRSA exhibited moderate diagnostic accuracy (AUC = 0.86). A recovery rate threshold of 59.5 yielded high sensitivity (100%) but limited specificity (67%) for identifying RSA-defined resistance.

**Conclusion:**

These findings confirm the continued expansion of the *PfK13* R561H lineage in Rwanda and provide a first *ex vivo* indication that the N490T variant confers an AR phenotype. While the eRRSA shows potential as a high-throughput screening tool for surveillance due to its high sensitivity, further refinement of recovery thresholds and clinical validation are required to improve its predictive specificity.

**Supplementary Information:**

The online version contains supplementary material available at 10.1186/s12936-026-06107-4.

## Background

Since the early 2000's, artemisinin combination therapies (ACTs) are the recommended first-line antimalarials for uncomplicated falciparum malaria. The ACTs combine a fast-acting artemisinin derivative with a partner drug of longer half-life, such as lumefantrine or amodiaquine. Since their introduction, the ACTs have significantly contributed to the declining malaria mortality in Sub-Saharan Africa (SSA), and they are a cornerstone of malaria control on the continent [1]. However, artemisinin partial resistance (AR) has now emerged in SSA and acutely threatens malaria control there [1]. In the past decade in Southeast Asia, AR has been defined by delayed parasite clearance in treated patients and increased ring-stage parasite survival after *ex vivo* or *in vitro* exposure to artemisinin derivatives [2–4]. In SSA, the validated and candidate markers of AR, i.e., mutations in the *Plasmodium falciparum Kelch 13* (*PfK13*) gene propeller domain [5], are predominately present in East Africa, particularly in Rwanda, Uganda, Democratic Republic of Congo, Eritrea, Ethiopia, South-Sudan, and Tanzania [6–15]. In addition, novel *PfK13* mutations of unknown significance are increasingly detected in East Africa (MARC SE-Africa dashboard [16]) necessitating their functional characterization to maintain meaningful molecular surveillance.

The standard for characterizing drug susceptibility of *PfK13* variants is the *ex vivo*/*in vitro* Ring-Stage Survival Assay (RSA) of *P. falciparum* [5], and its broad implementation forms a key pillar of the WHO strategy to respond to AR in Africa [1]. *Ex vivo* RSAs are applied on fresh clinical isolates, and *in vitro* RSAs on culture-adapted ones. In this process, young parasite ring stages are exposed to a pulse of dihydroartemisinin (DHA), allowed to recover for 66 h, and survival rates are estimated microscopically. This is a laborious, time-consuming, and multi-step assay, which requires experience and expertise, and which is difficult to scale. Replacing microscopy by quantitative PCR, the Extended Recovery Ring-Stage Survival Assay (eRRSA) promises a better scalability and accuracy of AR phenotyping [17, 18].

This work from Rwanda, a current epicenter of AR, presents AR phenotypes and *PfK13* genotypes of clinical *P. falciparum* isolates, collected in 2025. AR phenotypes were characterized by gold standard RSA and eRRSA, providing a comparison of both methods.

## Materials and methods

### Sample collection

The study was approved by the Rwanda National Ethics Committee (RNEC), reference RNEC/548/2024. Malaria patients were recruited in April 2025, in the Kirehe District of eastern province, Rwanda, respectively. Inclusion criteria were microscopically confirmed *P. falciparum* malaria, age above 1 year, and history of fever in the past 48 h. Following informed consent procedures, up to 5 mL venous blood was collected in EDTA, transported at room temperature for a maximum of 6 h, and stored at 4 °C for a maximum of 24 h before initiating drug susceptibility assays.

### *Ex vivo* RSA and eRRSA

Microscopy of Giemsa-stained thick blood films confirmed *P. falciparum* infection, and asexual parasites were counted on thin films *per* 2,000 erythrocytes. Samples with parasitemia of ≥0.1% and only ring-stage parasites were included for further testing. Following centrifugation, erythrocyte concentrates were washed twice in phosphate-buffered saline (PBS) including removal of the buffy coat. If parasitemia was >1%, it was adjusted to 0.5% by adding pre-washed uninfected O+ RBC. RSAs and eRRSAs were done as previously described [17, 18]. In brief, erythrocytes were diluted to obtain a 2% hematocrit in culture medium (RPMI-1640 with 2 mM L-glutamine and 25 mM HEPES [ThermoFisher Scientific], 0.2% NaHCO3, 10 mg/L gentamicin [Gibco], 0.1 mM hypoxanthine, and 0.5% AlbuMAX II [Gibco]), and plated out at 1 mL in each well, four wells per isolate. For each isolate, two wells were exposed to 700 nM dihydroartemisinin (DHA, dissolved in DMSO and further diluted in culture medium), two wells to 0.1% DMSO (i.e., the un-exposed control), and incubated by candle-jar method [19] for 6 h (37 °C, 3–5% CO_2_, 15–17% O_2_). After that, the erythrocyte pellets were washed twice with 20 mL culture medium, transferred to a new plate, and incubated again for an additional 66 h. After a total of 72 h, Giemsa-stained thin films were prepared from aliquots of the erythrocyte pellets, the culture medium was changed, and the plates were incubated again. After 120 h from the initial start of the assay, 20 μL of each assayed culture was stored at −20 °C for subsequent quantitative PCR (qPCR).

The *ex vivo* RSA growth rate of isolates at 72 h was determined microscopically as the parasitemia in un-exposed control wells divided by the initial parasitemia. The survival rate at 72 h was calculated as the parasitemia in DHA exposed wells divided by the parasitemia in unexposed control wells × 100. Microscopy was done by counting viable parasites *per* 10,000 erythrocytes. Duplicate values were averaged. The eRRSA read-out was done on the 120 h-samples by qPCR of the *PfCRT*-gene, with cycling conditions as previously described [17], on a Roche LightCycler 480. Cycle threshold (CT) values were calculated using the LightCycler 480 Software. The CTs for the two technical replicates for the untreated and treated samples were averaged. The fold-change in parasite growth (also called recovery rate) among exposed and non-exposed wells was determined by the difference between respective average CT values.

Assays were considered valid when the 72-h RSA growth rate was >1. Isolates were considered resistant when the survival rate was >1% in the *ex vivo* RSA.

### *PfK13* genotyping

DNA was extracted from the concentrated erythrocytes of the un-exposed control wells, after the eRRSA was completed (QIAamp DNA Blood Mini Kit, Qiagen, Germany). A single PCR for *PfK13* [5] generated amplicons (849 bp) that were sequenced by Sanger Sequencing (Eurofins genomics, Germany). Sequences were aligned to reference sequences from laboratory strain 3D7 (PlasmoDB.org), using CodonCode Aligner version 9.0.1 (CodonCode Cooperation), and mixed signals (defined as minor allele signal <20% of the major chromatogram peak) were considered as mutant.

### Statistical analysis

For 72-h survival rates (RSA) and for 120-h recovery rates in growth (eRRSA), we computed the median values and corresponding interquartile range. Both read-out values were tested for correlation with the initial parasitaemia by Spearman correlation analysis. We also did this after stratification by *PfK13* genotypes. We compared the survival rates/recovery rates between groups using Wilcoxon rank sum test, and both were compared for concordance by a Wilcoxon rank sum test and by Spearman correlation analysis. Spearman's Rho and corresponding p-values are reported where appropriate. To evaluate the screening accuracy of the eRRSA, we did a receiver operating characteristic (ROC) analysis. We generated an ROC curve, calculated the area under the curve, and selected an optimal cut-off for the 120-h recovery rate based on accuracy ((true positives + true negatives)/total). The RSA-based survival rate threshold of 1% was used as gold standard. We used R for all statistical analysis, including the package ROCR [20].

## Results

RSAs, eRRSAs, and sequencing were performed on 19 fresh patient isolates. About half of the isolates (52.6%, 10/19) carried a non-synonymous *PfK13* mutation, mostly the validated AR marker R561H (31.6%, 6/19). Other mutations were N490T (2, of which 1 in co-occurrence with R561H), P667S (2), and F699C (1).

**Fig. 1:**
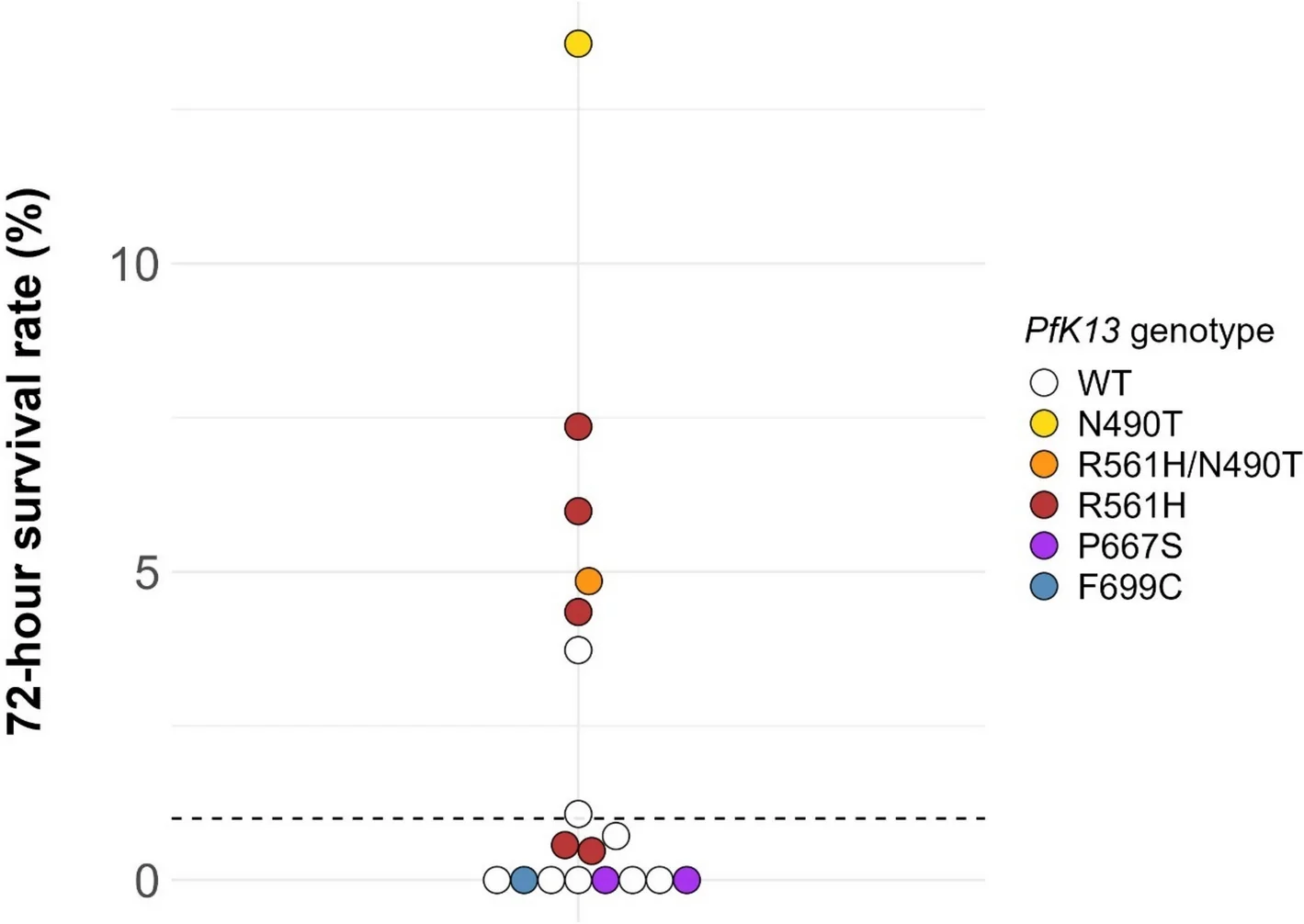
*Ex* *vivo* DHA susceptibility by RSA. Each point represents the result for a single isolate (N=18). The dashed line corresponds to the resistance threshold (72-h survival rate > 1%). Different colors correspond to different *PfK13* genotype

Eighteen isolates (94.7%) met the RSA validation criteria, and of these, 7 (38.9%) exhibited survival rates above 1% indicating AR (Fig. 1). The overall median 72-h RSA survival rate was 0.5% (interquartile range [IQR], 0.0–4.2). The survival rate did not associate with the initial parasitemia (ρ=0.2, *P*=0.3). For isolates with the validated AR marker R561H, the median survival rate was 4.6% (IQR, 1.5–5.7, n=6), which was significantly higher than in wildtype isolates (0.0% [IQR, 0.0–0.8], n=8, *P*=0.02). Likewise, the two isolates with N490T had higher survival rates: 13.6% and 4.8% (the latter in co-occurrence with *PfK13* R561H). For isolates carrying *PfK13* P667S, survival rates were each 0. Parasite survival rates in isolates with any *PfK13* mutation (validated, candidate, or other non-synonymous mutations) were higher although not significantly (2.5 [0.1–5.7], n=10) than wildtype isolates (*P*=0.1).

**Fig. 2:**
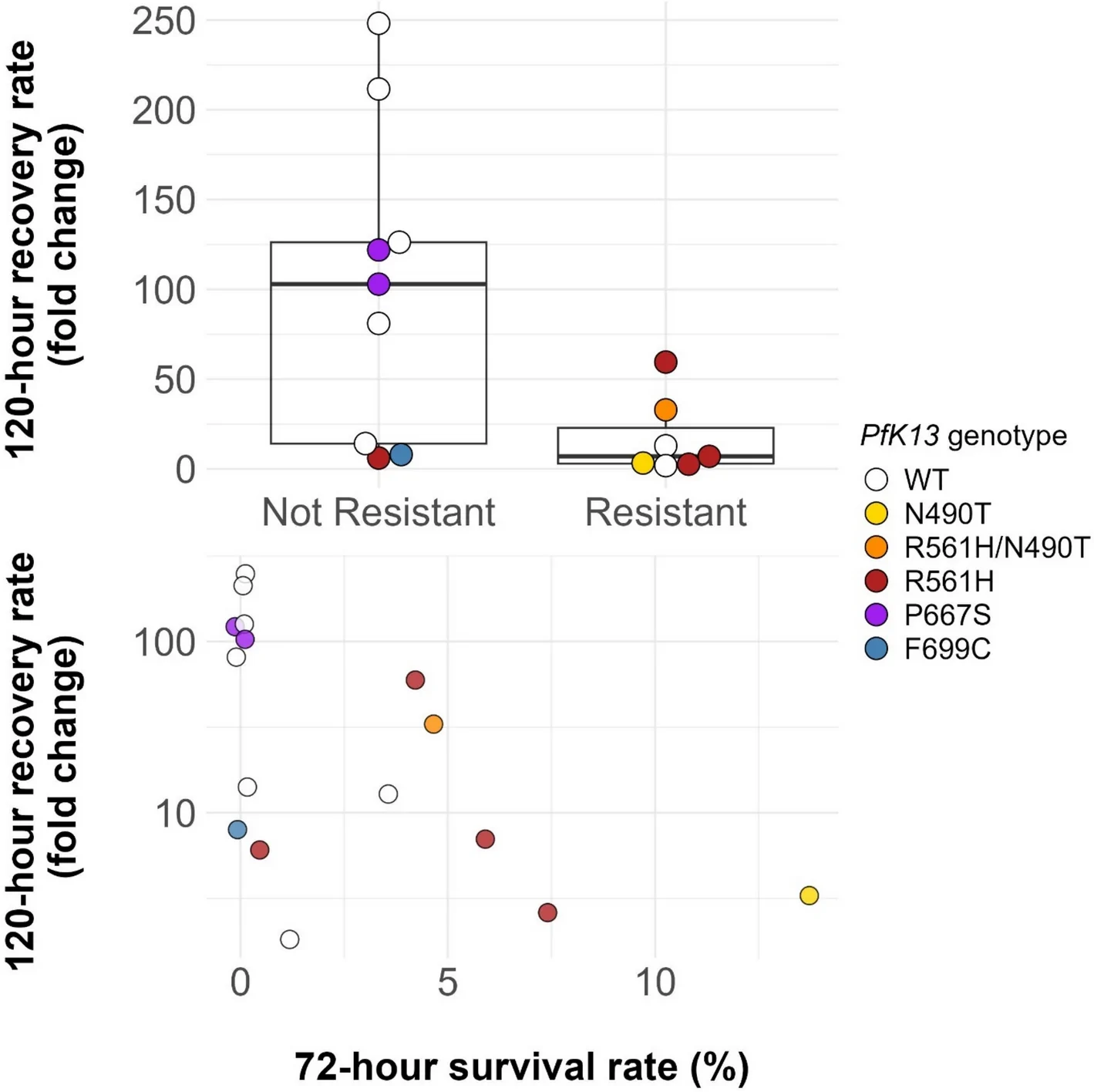
Upper: eRRSA recovery rates, distinguished by RSA profile (Not resistant: survival rate <1%; Resistant: survival rate >1%). Center line of boxes correspond to the median, and minimal and maximal bounds correspond to 25th and 75th percentiles, respectively. Lower: Correlation between RSA and eRRSA results. Each point represents the result for a single isolate (N=16). Different colors correspond to different *PfK13* genotype. Jitter (0.2%) was added to the x-axis values to visualize overlapping points

Regarding eRRSA, 16 assays met the validation criteria (complete data, 72 h growth rate >1%; 120 h recovery rate of fold-change >1), and each of these assays also met the RSA validation criteria (Fig. 2). The overall median recovery rate among the eligible assays was a fold change of 23.5 (IQR, 6.8–107.7). The recovery rate did not significantly associate with the initial parasitemia (ρ=−0.5, *P*=0.07). RSA survival rates and eRRSA recovery rates were correlated (ρ=−0.7, *P*=0.003; Fig. 2, lower panel). However, the median eRRSA recovery rate did not differ between isolates harbouring the validated AR marker R561H and wildtype isolates (7.0 [IQR, 6.1–32.9], n=5 *vs* 81.0 [13.5–168.9], n=7, *P*=0.2). Isolates passing the resistance threshold in the RSA, had a lower recovery rate in the eRRSA as compared to non-resistant isolates (7.0, [2.9–22.9], n=7 vs 102.9 [14.1–126.2], n=9, *P*=0.02, Fig. 2, upper panel).

**Fig. 3:**
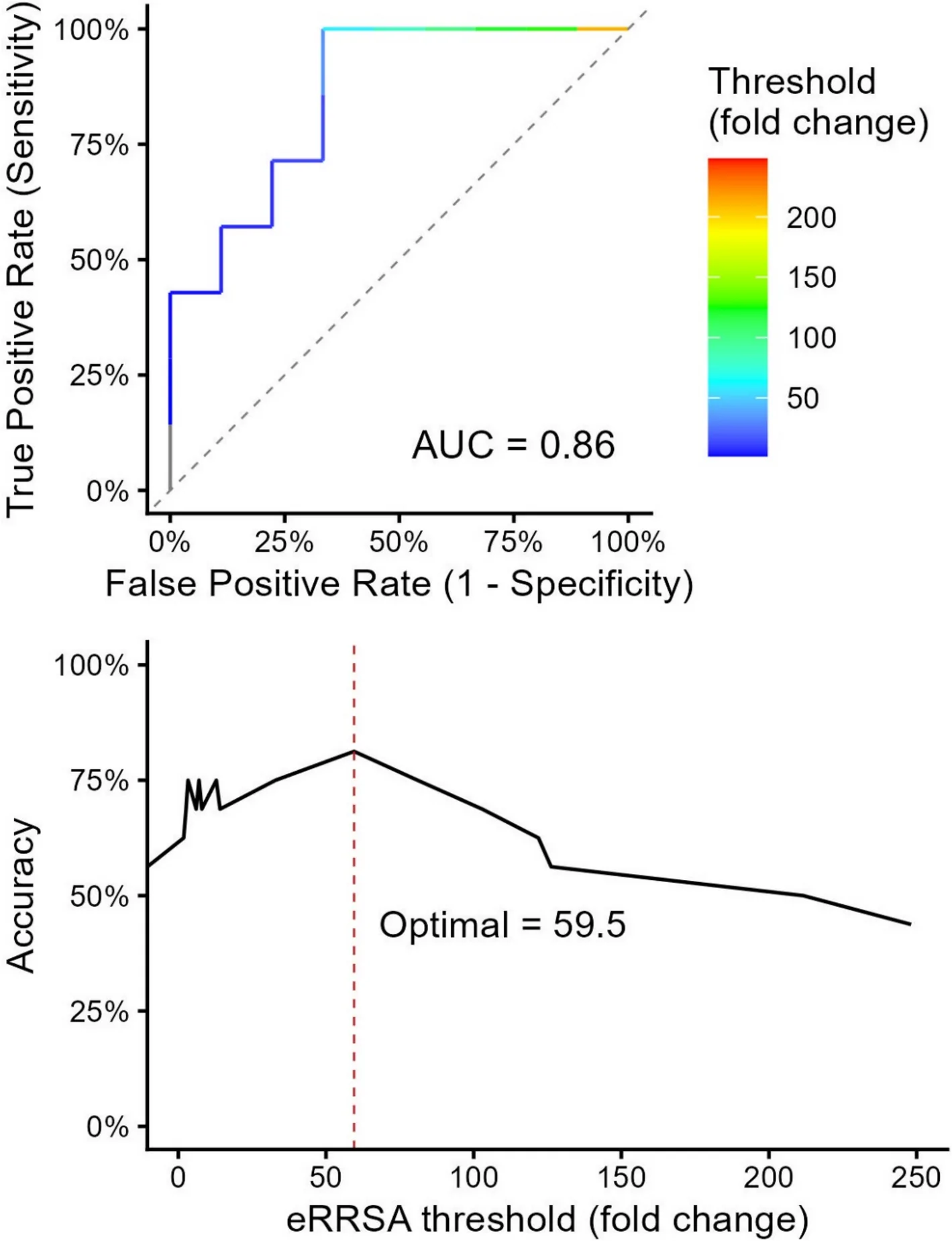
Upper: ROC curve for predicting partial artemisinin resistance in *Plasmodium falciparum* patient isolates, as defined by *ex* *vivo* RSA, using the eRRSA read-out value for recovery rate. The colored bar on the right side indicates cut-off (threshold) values. Lower: Accuracy by different cut-off values of eRRSA read-out value for recovery rate

The screening performance of the eRRSA to detect RSA-defined AR was evaluated by ROC analysis (Fig. 3). The area under the curve of the ROC was 0.86, corresponding with a medium screening performance for the eRRSA read-out in our sample set (a value of 1 would perfectly distinguish between positive and negative cases, 0.5 would be similar to random guessing) (Fig. 3, upper panel). A 120-h recovery rate threshold of 59.5 was selected based on Youden Index (sensitivity + specificity−1, maximized) in our sample set, reaching an accuracy of 0.86 (Fig. 3, lower panel). This threshold of 59.5 would allow us to correctly identify 100% of the resistant isolates (as defined by *ex vivo* RSA) and would falsely identify 33% of sensitive isolates as resistant, corresponding to a positive predictive value of 70%.

## Discussion

The emergence of AR in East Africa emphasizes the need to strengthen capacity for monitoring antimalarial drug resistance, both by molecular surveillance and by functional characterization. We show that in 2025, around 30% of isolates harbor the validated R561H marker for AR, and thus, a continuous increase in this variant in Rwanda [14]. In addition, for the first time in Rwanda we report the N490T polymorphism in two isolates [16]. This variant was previously seen in western Uganda at 10% prevalence [21]. So far of unknown significance, we here show that parasites with *PfK13* N490T may have an *ex vivo* RSA survival rate indicating AR (in our data, a 72 h survival rate of 13.6). *PfK13* P667S has previously been reported in a Rwandan patient with delayed parasite clearance [22] and in one out of 783 isolates in Kenya [23]. Our *ex vivo* RSA data on isolates harboring P667S do not suggest AR resistance. *PfK13* F699C has recently also been observed once among 87 isolates from Rukara, eastern Rwanda [24], some 50 km north of Kirehe, where our one respective isolate originated from. Based on the *ex vivo* RSA data of this one isolate, it seems unlikely that the variant confers AR. Two isolates without *PfK13* mutations exhibited an *ex vivo* phenotype indicative of AR in both assays. This is unlikely to reflect inadequate DHA exposure during the assays, as other isolates processed in the same experimental batches demonstrated the expected sensitive phenotype. However, a limitation of our experimental design is the absence of susceptible control isolates. Potentially, other genetic markers are responsible for this AR signature, such as mutations in *PfCoronin* [25, 26], and the PIN-haplotype in *PfPX1* [27, 28], and a comprehensive genomic screening is warranted.

Similar to other studies [17, 18], our eRRSA and *ex vivo* RSA data correlated, even if only weakly. In the present study, eRRSA outputs did not suggest AR for all the isolates with the validated AR marker R561H. Nevertheless, this assay proved to be sensitive to detect isolates with increased survival in the *ex vivo* RSA. Specificity and predictive value were limited. The low sample size of the present study, a significant limitation, might be responsible for that. The intended purpose of the eRRSA is essential in defining an appropriate threshold for AR. For high-throughput AR surveillance in non-affected areas prioritizing high sensitivity of an assay to detect AR may be preferable at the expense of specificity. The initial study presenting the eRRSA used *in vitro* culture on adapted and cloned south-east Asian *P. falciparum* isolates [17]. In that study, eRRSA read-outs correlated closely with parasite clearance times in treated patients. *Ex vivo* RSA read-outs on clinical isolates may present with more noise, possibly because of the presence of multiclonal infections and because of the variable developmental stage of the parasite’s ring form after sample storage. Our *ex vivo* results suggest an eRRSA recovery rate threshold of approximately 50 to detect AR (as defined by RSA), but most relevant would be to define accuracy for predicting patient parasite clearance or treatment outcome, which are both parameters not included in this study. Investigating the clinical implication of increased survival in *ex vivo* assays should be prioritized in currently affected populations to refine threshold values.

While the eRRSA introduces PCR-based quantification as an additional step, this is offset by the gains in scalability that molecular readout confers over microscopic counting, both in the number of isolates processed in parallel and in the inclusion of technical replicates for robust outcomes [17]. The 48-h culture extension amplifies the phenotypic difference between resistant and sensitive parasites by allowing a full additional intraerythrocytic cycle for recovery, improving both discriminatory power and assay consistency [17]. This extended timepoint combined with molecular readout also avoids the challenge of distinguishing pyknotic from viable parasites at the 72-h microscopic readout, a source of significant inter-observer variability in the standard RSA. The eRRSA qPCR adds reagent and equipment costs compared to microscopy, although the reduction in skilled microscopist time may counterbalance this, particularly when scaled.

In summary, we show a further increase of the validated AR marker *PfK13* R561H in Rwanda [14, 15], the presence of N490T in this country and its *ex vivo* indication for AR, and the lack of *ex vivo* AR indication for two rare, previously detected mutations. These data illustrate the necessity to further expand molecular and *ex vivo* assays for monitoring AR in East Africa.

## Supplementary Information

Supplementary Material 1

## Data Availability

The datasets used and analysed for the current study is available is supplementary material to this manuscript.
